# The influence of the level of physical activity and human development in the quality of life in survivors of stroke

**DOI:** 10.1186/1477-7525-9-89

**Published:** 2011-10-13

**Authors:** Felipe J Aidar, Ricardo J de Oliveira, António J Silva, Dihogo G de Matos, André L Carneiro, Nuno Garrido, Robert C Hickner, Victor M Reis

**Affiliations:** 1 Department of Sports Science, Exercise and Health of the Trás-os-Montes e Alto Douro University, Vila Real, Portugal; 2 University of Brasília-Unb, Brasília, Distrito Federal, Brazil; 3 Human Performance Laboratory, Department of Exercise and Sport Science, and Department of Physiology, East Carolina University. Greenville, NC, USA

**Keywords:** Cerebrovascular Accident, Physical Activity, Stroke, Quality of Life

## Abstract

**Background:**

The association between physical activity and quality of life in stroke survivors has not been analyzed within a framework related to the human development index. This study aimed to identify differences in physical activity level and in the quality of life of stroke survivors in two cities differing in economic aspects of the human development index.

**Methods:**

Two groups of subjects who had suffered a stroke at least a year prior to testing and showed hemiplegia or hemiparesis were studied: a group from Belo Horizonte (BH) with 48 people (51.5 ± 8.7 years) and one from Montes Claros (MC) with 29 subjects (55.4 ± 8.1 years). Subsequently, regardless of location, the groups were divided into Active and Insufficiently Active so their difference in terms of quality of life could be analyzed.

**Results:**

There were no significant differences between BH and MCG when it came to four dimensions of physical health that were evaluated (physical functioning, physical aspect, pain and health status) or in the following four dimensions of mental health status (vitality, social aspect, emotional aspect and mental health). However, significantly higher mean values were found in Active when compared with Insufficiently Active individuals in various measures of physical health (physical functioning 56.2 ± 4.4 vs. 47.4 ± 6.9; physical aspect 66.5 ± 6.5 vs. 59.1 ± 6.7; pain 55.9 ± 6.2 vs. 47.7 ± 6.0; health status 67.2 ± 4.2 vs. 56.6 ± 7.8) (arbitrary units), and mental health (vitality 60.9 ± 6.8 vs. 54.1 ± 7.2; social aspect 60.4 ± 7.1 vs. 54.2 ± 7.4; emotional aspect 64.0 ± 5.5 vs. 58.1 ± 6.9; mental health status 66.2 ± 5.5 vs. 58.4 ± 7.5) (arbitrary units).

**Conclusions:**

Despite the difference between the cities concerning HDI values, no significant differences in quality of life were found between BH and MCG. However, the Active group showed significantly better results, confirming the importance of active lifestyle to enhance quality of life in stroke survivors.

## Background

Indicators related to health have assumed a prominent position in the measurement of human development. The incidence of disease in many countries has significantly increased in recent years [1-4]. Stroke currently occupies the third position with regard to mortality, being also the leading cause of disability in Western countries and ranking first in terms of loss of quality adjusted life years [1-3]. Moreover, problems related to hemiplegia, hemiparesis and aphasia occur frequently in stroke victims, and cause direct and indirect financial impacts on the public health system [4,5]. In Brazil, the disease accounts for one third of the deaths from circulatory diseases per year, and has a prominent place next to cardiovascular diseases [6].

There is epidemiological evidence that physical activity may lower mortality due to cardiovascular events, which could be explained by a lower blood pressure, an increased HDL-C and a lower incidence of diabetes in physical active people [7]. Additionally, studies have shown that physical activity attenuates the re-incidence of strokes and improves the quality of life (QOL) post-stroke [8,9].

Despite the proven positive impact of physical activity on QOL, analysis of this relationship within a framework related to the Human Development Index (HDI) is absent in the literature. There has always been a worldwide concern to measure the levels of human development. Thus, in early 1990, the HDI was created by the United Nations (UN). The HDI is a multifaceted index of human development that is based on economic indicators as well as indicators related to education and longevity. The HDI is the best known human development index [1].

Thus, this study aimed to identify differences in physical activity level and in the quality of life of stroke survivors in two cities differing in human development index ranking in Brazil.

## Methods

### Sample

The study included, initially, 77 people: 48 from the Metropolitan Region of Belo Horizonte (BH: Brazil HDI ranking of 80), Minas Gerais, Brazil (BHG) and 29 from Montes Claros (MC: Brazil HDI ranking of 968), Minas Gerais, Brazil (MCG) (Table 1). Later the subjects were divided into two groups (Active, and Insufficiently Active) based on the amount of physical activity performed (Table 2), regardless of the place of residence. The groups were composed of people who voluntarily sought a physical activity program offered by the Firefighters of Minas Gerais. The program assists people with special needs and organizes physical activities.

**Table 1:** Age of participants in groups sorted by HDI of the cities

Belo Horizonte Group	Ocurrence/Means ± SD
Age (yr)	51.5 ± 8.7
Age-male (yr)	50.7 ± 7.8
Age-female (yr)	52.3 ± 6.8
Gender (Men/Women) (%)	18 (37.5)/30 (62.5)

Montes Claros Group	Ocurrence/Means ± SD

Age (yr)	55.4 ± 8.1
Age-male (yr)	53.2 ± 9.6
Age-female (yr)	57.6 ± 7.7
Gender (Men/Women) (%)	11 (37.9)/18 (82.1)

**Table 2:** Age of participants in groups sorted by level of physical activity

Active	Ocurrence/Means ± SD
Age (yr)	56.1 ± 6.1
Age-male (yr)	54.8 ± 8.3
Age-female (yr)	57.4 ± 5.7
Gender (Men/women) (%)	6 (28.6)/15 (71.4)

Insufficiently Active	Ocurrence/Means ± SD

Age (yr)	50.8 ± 8.1
Age-male (yr)	50.1 ± 6.8
Age-female (yr)	51.5 ± 5.9
Gender (Men/women) (%)	20 (35.7)/36 (64.3)

The eligibility criteria consisted of the individual having suffered a stroke at least one year prior to the study and showing sequelae, hemiplegia or hemiparesis.

The individuals were classified according to the Rankin Scale [10,11], and in the BHG 33.1% had mild disability, 44.2% showed moderate disability and 22.7% higher disability. In the MCG 26.4% had mild disability, 48.5% showed moderate disability and 22.1% higher disability. In a second classification using the same scale, for the Active group, 29.7% individuals had mild disability, 50.8% showed moderate disability and 19.5% higher disability. For the Insufficiently Active group 24.4% had mild disability, 47.8% moderate disability and 27.8% higher disability.

All volunteers were informed about the study and signed a consent document in accordance with the Declaration of Helsinki (1964, revised in 1975, 1983, 1989, 1996 and 2000). The procedures were approved by the institution ethics committee.

#### Procedures

Data collection was performed at the time of registration for the program, and all interviews were conducted by a single experienced social services technician. The interviews evaluated the HDI, the QOL SF36 and the IPAQ, as described below.

### Instruments

#### Human Development Index-HDI

The HDI seeks to demonstrate the success achieved in three basic human needs: access to knowledge (education), the right to a long and healthy life (longevity) and the right to a decent standard of living (income).

Regarding education (HDI-E), the indicators are the literacy rate of the population over 15 years and the proportion of people with access to primary, secondary and higher education. When it comes to longevity (HDI-L), the indicator is represented by life expectancy, whereas the indicator for income (HDI-I) is represented by the GDP per capita, which synthesizes the population's capacity to purchase goods and services, thus tending to represent the access to other dimensions not covered by the HDI.

The process of HDI determination consists of choosing upper and lower parameters for each indicator and the normalization is given by an equation that measures the distance between the observed value for the indicator and the minimum value as a proportion of the distance between the maximum and minimum value where I = (observed value-minimum value)/(maximum value-minimum value). Thus, the closer the observed value is to the minimum value, the smaller is the numerator and therefore the worse the situation of development. Conversely, the closer the observed value is to the maximum value, the better the situation in terms of development.

The maximum and minimum values are defined through the observation of the world tendencies in terms of indicators for the long term, allowing periodic comparisons of the indexes obtained.

It is noteworthy that there are 27 states in Brazil, and that the HDI Table is based on the territorial division in 2000, year of completion of the last census, with 5,507 cities. The periods of interest for this study, on the other hand, correspond to the years until 2010, when the number of municipalities in the National Territory was already 5,564. Therefore, the absence of HDI was verified for 57 new municipalities. However, Minas Gerais (the state targeted by the present study) continued in the period with 853 municipalities; thereby showing no change in its geopolitical scenario (see table 3).

**Table 3:** HDI and development indicators in the cities of reference

City	LEB	PCI	HDIM-L	HDI-E	HDI-I	HDI-M	NR
Belo Horizonte	70.520	557.435	0.759	0.929	0.828	0.839	80
Montes Claros	72.247	245.425	0.787	0.872	0.691	0.784	968

**Source: **Minas Gerais. João Pinheiro Foundation. United Nations (UN) [19]*

Abbreviations:

HDIM-L: Municipal Human Development Index-Longevity; LEB: Life Expectancy at Birth; PCI: Per Capita Income; HDI-E: Education Index; HDI-I: Gross Domestic Income; HDI-M: Municipal Human Development Index; NR: National Ranking

*This publication contained only descriptive data of HDI.

In this sense, both the HDI and its three sub-indices (HDI-E, HDI-L and HDI-I) vary (arbitrary units) between 0 and 1, being classified as 0 to 0.5 low human development, from 0.5 to 0.8 medium human development and 0.8 to 1, high human development [12].

#### Generic Questionnaire for the Assessment of Quality of Life SF 36, Health Research

The "Generic Questionnaire for the Assessment of Quality of Life"-SF 36 "Health Research", previously validated [13-16], consists of 36 questions, ten of which related to functional capacity, four to physical aspects, two related to pain, five to health status, four to vitality, two associated with social aspects, three with emotional aspects, five with mental health, and one related to current health condition and one year after stroke [17].

The questionnaire is based on a review of existing instruments, taking into account functional changes and limitations, as well as social aspects [14]. The scores range from zero to 100 (arbitrary units), with higher scores indicating better quality of life.

#### "International Physical Activity Questionnaire"-IPAQ

In order to verify the level of physical activities performed, the International Physical Activity Questionnaire-IPAQ was used in its short version, containing objective questions regarding the frequency and duration of the physical activities. Individuals were classified as very active, active, irregularly active and sedentary [18].

#### IPAQ Classification

The IPAQ contained questions about activities performed during the week prior to the questionnaire. The subjects' data were tabulated, evaluated and ranked according to the IPAQ, which divides and conceptualizes categories as follows:

**- Sedentary: **Not performing any physical activity for at least 10 continuous minutes during the week;

**- Insufficiently active: **individuals who practice physical activities for at least 10 continuous minutes per week, but insufficiently to be classified as active. For the classification of the individuals under this category, the length and frequency of the different types of activities (hiking + moderate + vigorous) were taken into account. This category is divided into two groups:

**- Insufficiently Active A: **Performing 10 minutes of continuous physical activity, following at least one of the above criteria: frequency 5 days/week or duration-150 minutes/week;

**- Insufficiently Active B: **Not meeting any of the criteria adopted for Insufficiently Active A;

**- Active: **Meets the following recommendations: a) vigorous physical activity-> 3 days/week and > 20 minutes/session; b) moderate or walking-> 5 days/week and > 30 minutes/session; c) any activity added > 5 days/week and > 150 min/week;

**- Very Active: **Meets the following recommendations: a) vigorous-> 5 days/week and > 30 min/session; b) vigorous-> 3 days/week and > 20 minutes/session + moderate or walking 3-5 days/week and > 30 minutes/session.

For the study, very active and active individuals were classified as Active, whereas insufficiently active and sedentary subjects were placed in the Insufficiently Active group.

#### Statistics

Statistical analysis was done with the groups (BHG and MCG) and the homogeneity of the sample was verified through the Shapiro Wilk test. Given the non-normality of the sample, the Mann-Whitney test was used for checking the difference in quality of life in different cities, as well as for checking the difference in quality of life between active and insufficiently active groups.

We adopted a p < 0.05, and the data analysis was carried out using SPSS for Windows version 15.0.

## Results

The values for HDI are described in Table 3. It is noteworthy that both Belo Horizonte and Montes Claros are in the state of Minas Gerais, which has the second best performance in the country in economic terms. However, Montes Claros has only a moderate HDI-I economic indicator (0.691), a per capita income of 245,425, and a national HDI ranking of 968; whereas Belo Horizonte has a high HDI-I economic indicator (0.828), a per capita income of 557,435, and a national HDI ranking of 80.

Patients coming from the cities of Belo Horizonte and Montes Claros were evaluated with respect to quality of life (Table 4).

**Table 4:** Measures of physical and mental health according to the SF 36 Questionnaire in groups

	BH	MCG	P
**Physical Health**			

Functional capacity	49.1 ± 6.0	47.5 ± 7.9	0,706
Physical Aspects	61.3 ± 6.7	59.4 ± 7.7	0,804
Pain	48.2 ± 6.2	50.1 ± 6.0	0,077
General Health Status	58.8 ± 7.9	59.3 ± 6.8	0,913

**Mental Health**			
Vitality	56.5 ± 7.6	55.2 ± 8.2	0,638
Social Aspects	55.2 ± 6.6	54.5 ± 8.4	0,103
Emotional Aspects	58.4 ± 5.4	59.3 ± 7.9	0,079
Mental Health	61.2 ± 4.5	59.1 ± 8.5	0,051

* p < 0,05 (Mann-Whitney). Data are presented as mean ± SD.

There were no statistically significant differences between the quality of life in BH and MC, which are cities with different HDI.

Subsequently, the level of quality of life in all participants was evaluated in relation to the amount of physical activity performed, according to the SF 36 questionnaire, regardless of location (Table 5).

**Table 5:** Measures of physical and mental health in the Active and the Insufficiently Active Group

	Active	Insufficiently Active	P
**Physical Health**			

Functional Capacity	56.2 ± 4.4*	47.4 ± 6.9	0,036
Physical Aspects	66.5 ± 6.5*	59.1 ± 6.7	0,042
Pain	55.9 ± 6.2*	47.7 ± 6.0	0,035
General Health Status	67.2 ± 4.2*	56.6 ± 7.8	0,003

**Mental Health**			

Vitality	60.9 ± 6.8*	54.1 ± 7.2	0,038
Social Aspects	60.4 ± 7.1*	54.2 ± 7.4	0,036
Emotional Aspects	64.0 ± 5.5*	58.1 ± 6.9	0,022
Mental Health	66.2 ± 5.5*	58.4 ± 7.5	0,012

* p < 0,05 (Mann-Whitney). Data are presented as mean ± SD

## Discussion

This study aimed to identify differences in physical activity level and in the quality of life of stroke survivors in two cities differing in economic aspects of the human development index. The main findings of the present study were that factors such as location and socioeconomic issues cannot be considered, *de per si*, indicators of quality of life, and that physical activity plays an important role in improving quality of life, regardless of the HDI-I economic indicator or HDI national ranking status of the city of residence.

When evaluating the indicators of HDI in the two cities, significant differences were found with regard to per capita income (245.425 R$ in MC and 557.435 R$ in BH), HDI-I, which represents the gross domestic product (0.691 in MC and 0.828 in BH) and the position of the municipalities in the Brazilian HDI ranking (968^th ^place for MC and 80^th ^place for BH). However, other indicators showed no major differences, such as the education-related HDI-E, which was 0.929 in BH and 0.872 in MC. It is noteworthy that Montes Claros, although economically poorer, has a higher life expectancy at birth (LEB) than does Belo Horizonte (72.242 yrs in MC and 70.520 yrs in BH) [12]. Contrary to our findings that the economic indicators of HDI were not decisive determinants of quality of life in survivors of stroke, another study found that socioeconomic conditions and difficulties in accessing health services tend to expose people to an increased risk of death [20,21].

One possible explanation for stroke survivorship and quality of life, regardless of economic indicators in HDI, can be attributed to the fact that there is now a universalized health system in Brazil. The Brazilian health care system now offers more standard treatments, more accurate diagnoses, and acts within a time considered optimal, i.e. within three hours after the stroke and with the most appropriate procedure [22-29]. In recent years there has been a downward trend in cerebrovascular problems, which allowed Brazilian indices to be close to those seen in countries like the U.S. and Canada. This is a reflection of improvements in public health policies pursued in recent years [28].

Diagnosis and treatment carried out in the early stages of stroke tend to decrease the length of stay in hospital, improve patient prospects and decrease chances of permanent sequelae. These findings from our previous study [28] leads us to believe that economic standing seems not to be a determining factor in quality of life. This explains the absence of significant differences in quality of life in patients affected by stroke in Belo Horizonte and Montes Claros, who were subjected to similar treatment despite the aforementioned economic differences (HDI-R BH = 0.828 and MC = 0.691). On the other hand, delayed treatment may be more difficult and leave sequelae, regardless of location [30].

Comparing our results with those of other studies, research conducted in Chile with patients who had suffered a stroke more than three years before showed better results than those found in our study in terms of quality of life. In that study, 59 patients (average age of 62 and 51% female) were evaluated. Nearly half (n = 29: 49.1%) of the patients had some limitation. Thirty patients (50.9%) presented with functional independence, despite showing minimal sequelae. The group of stroke survivors (aged 20-36 years) achieved an average of 84 points, and the older group (average age of 75 years) achieved an average quality of life score of 63 points [31].

It is believed that changes in lifestyle play a major role in the prognosis of patients who suffered stroke [32,33]. Likewise, a study conducted in Japan showed that physical activity tends to reduce the risk of mortality in patients who had stroke [34]. Physical activity is also believed to be beneficial in cases of ischemic stroke, reducing the area of ischemia in animals [35-37]. Although caution should be taken when extrapolating results from animal studies to humans, it appears that physical activity may lessen the severity of stroke, improving quality of life and the capacity for work and leisure, thereby reducing the chances of new strokes and the functional imitations they can bring [34,36]. In this sense, physical activities can be considered the best way to improve the psychosocial indicators, quality of life and stress levels in people with physical limitations, bringing about improvements in social and emotional health [9].

Additionally, physical exercises are a major method of reducing stress and improving social and emotional well-being in people with physical limitations [9]. Specifically in relation to stroke survivors, daily physical activity can improve quality of life as confirmed in a previous study of 40 stroke survivors. We assessed participation in daily physical activity post stroke in relation to quality of life and health. The results suggest that daily physical activity on an outpatient basis is associated with a better quality of life and health in stroke survivors [38]. Similar results were obtained in a study of stroke survivors subjected to water activities [8]. Quality of life was better in the physically active stroke survivors than those who were not active [8].

## Conclusion

We therefore conclude that factors such as location and socioeconomic issues cannot be considered, *de per si*, indicators of quality of life, and that physical activity plays an important role in improving quality of life, regardless of the HDI ranking and economic status of the city of residence.

## Competing interests

The authors declare that they have no competing interests.

Felipe José Aidar had no contracts or financial dealings with any company related to this manuscript. Ricardo Jacó de Oliveira, António José Silva, Dihogo Gama de Matos, Nuno Garrido, André Luiz Carneiro, Robert C. Hickner and Victor Machado Reis have no consultant ships, company holdings or patents. This study did not receive any financial support for research. All authors any direct or indirect conflicts of interests related to this manuscript. All authors meet the criteria for authorship stated in the Uniform Requirements for Manuscripts Submitted to Health and Quality of Life Outcomes.

## Authors' contributions

FJA and DGM conceived the study. NG, ALC, AJS participated in data collections of the study, VR conducted data analysis. RJO and RCH participated in interpretation of data and manuscript preparation. All of the authors reviewed the manuscript prior to submission.
